# Molecular Epidemiology and Phylodynamics of the Human Respiratory Syncytial Virus Fusion Protein in Northern Taiwan

**DOI:** 10.1371/journal.pone.0064012

**Published:** 2013-05-29

**Authors:** Hsin Chi, Hsin-Fu Liu, Li-Chuan Weng, Nai-Yu Wang, Nan-Chang Chiu, Mei-Ju Lai, Yung-Cheng Lin, Yu-Ying Chiu, Wen-Shyang Hsieh, Li-Min Huang

**Affiliations:** 1 Department of Pediatrics, Mackay Memorial Hospital, Taipei, Taiwan; 2 Department of Medical Research, Mackay Memorial Hospital, Tamshui, Taiwan; 3 Mackay Medicine, Nursing and Management College, Taipei, Taiwan; 4 Graduate Institute of Clinical Medicine, National Taiwan University College of Medicine, Taipei, Taiwan; 5 Department of Pediatrics, National Taiwan University Hospital, Taipei, Taiwan; 6 Institute of Bioscience and Biotechnology, National Taiwan Ocean University, Keelung, Taiwan; 7 Department of Laboratory Medicine, Mackay Memorial Hospital, Taipei, Taiwan; 8 Department of Laboratory Medicine, Taipei Medical University-Shuang Ho Hospital, New Taipei City, Taiwan; 9 School of Medical Laboratory Science and Biotechnology, Taipei Medical University, Taipei, Taiwan; 10 Graduate Institute of Biomedical Informatics, Taipei Medical University, Taipei, Taiwan; University of Georgia, United States of America

## Abstract

**Background and Aims:**

The glycoprotein (G protein) and fusion protein (F protein) of respiratory syncytial virus (RSV) both show genetic variability, but few studies have examined the F protein gene. This study aimed to characterize the molecular epidemiology and phylodynamics of the F protein gene in clinical RSV strains isolated in northern Taiwan from 2000–2011.

**Methods:**

RSV isolates from children presenting with acute respiratory symptoms between July 2000 and June 2011 were typed based on F protein gene sequences. Phylogeny construction and evaluation were performed using the neighbor-joining (NJ) and maximum likelihood (ML) methods. Phylodynamic patterns in RSV F protein genes were analyzed using the Bayesian Markov Chain Monte Carlo framework. Selection pressure on the F protein gene was detected using the Datamonkey website interface.

**Results:**

From a total of 325 clinical RSV strains studied, phylogenetic analysis showed that 83 subgroup A strains (RSV-A) could be further divided into three clusters, whereas 58 subgroup B strains (RSV-B) had no significant clustering. Three amino acids were observed to differ between RSV-A and -B (positions 111, 113, and 114) in CTL HLA-B*57- and HLA-A*01-restricted epitopes. One positive selection site was observed in RSV-B, while none was observed in RSV-A. The evolution rate of the virus had very little change before 2000, then slowed down between 2000 and 2005, and evolved significantly faster after 2005. The dominant subtypes of RSV-A in each epidemic were replaced by different subtypes in the subsequent epidemic.

**Conclusions:**

Before 2004, RSV-A infections were involved in several small epidemics and only very limited numbers of strains evolved and re-emerged in subsequent years. After 2005, the circulating RSV-A strains were different from those of the previous years and continued evolving through 2010. Phylodynamic pattern showed the evolutionary divergence of RSV increased significantly in the recent 5 years in northern Taiwan.

## Introduction

Human respiratory syncytial virus (RSV) infection causes bronchiolitis and pneumonia and is the leading cause of hospitalization of infants and young children [1]–[6]. RSV, a member of the *Paramyxoviridae* family, is an enveloped virus with a nonsegmented, single-stranded, negative sense RNA genome [1], [7]. The most important antigenic proteins include the attachment glycoprotein (G protein) and the fusion glycoprotein (F protein), which mediate attachment to host cells and viral penetration, respectively [7]. Based on the G protein, RSV is classified into 2 major subgroups, RSV-A and RSV-B, and the both subgroups are further classified into genotypes based on genetic divergence [8], [9]. Antigenic variation between and within the two RSV subgroups may contribute to repeated RSV infections in an individual and the occurrence of annual epidemics by the evasion of pre-existing host immune responses to the G protein [9]. The amino acid variability in the G protein might be due to positive selection [10].

RSV F protein is synthesized as a precursor, F0, which is activated after being cleaved at two sites by furin-like intracellular host protease. The process yields an N-terminal F2 subunit, p27, and a C-terminal; membrane-anchored F1 subunit carrying the fusion peptide [7] and the F1 subunit is more conserved than F2 subunit [11]. The RSV F protein is highly conserved between subgroups A and B [7], [11] and is the principal antigen responsible for the protective immune response [12]. Virus-specific cytotoxic T lymphocytes (CTLs) play a major role in the clearance of RSV infection, and several studies indicate that the F protein is a target for CTLs [13]–[15]. Two related human HLA class I-restricted epitopes, HLA-A*01 (109–118) [13] and HLA-B*57 (106–114) [14] have been identified and the CTL epitopes are subgroup A-specific [16]. The F protein is thus important to understand whether immune selection over consecutive epidemics occurs in its epitopes recognized by neutralizing antibodies and CTLs and of interest in the development of an RSV vaccine.

Phylogenetic analysis of RSV G protein in annual epidemics has demonstrated that both subgroups can circulate concurrently. Switching of the predominant genotypes occurs and it might be determined by local factors, especially the level of herd immunity to certain strains [17], [18]. Phylodynamic analyses of viral genetic data are increasingly used to elucidate how evolutionary and ecological processes can jointly drive fluctuations in the genetic diversity of viral populations and how the virus escapes host immune responses [19], [20]. Although the molecular epidemiology and evolutionary dynamics of RSV have been studied, most studies are one the G protein gene [21], [22]. The F protein is the major focus regarding prophylactic antibodies, and for subunit or recombinant vaccine research [23]–[25]. The antigenic variation and the evolutionary patterns of the F protein genes are still not known. We investigated the phylogenetic relationship, evolutionary variability, CTL epitopes and population dynamics of the RSV F protein gene in northern Taiwan throughten consecutive years.

## Materials and Methods

### Ethics Statement

Ethical approval for this study was obtained from the Mackay Memorial Hospital Ethics Committee (IRB number: MMH-I-S-627, protocol title: Clinical Features of Pediatric Respiratory Syncytial Virus Infections: Risk Factors and outcome). We retrospectively collected the demographic data including length of fever and hospital stay, oxygen use and laboratory results from the charts. Since the data were collected from the patients, who received regular medical management, by retrospective review of medical charts, a written informed consent from the patients was waived.

### Patients and Samples

RSV were obtained from virus stocks collected between July 2000 and June 2011 from nasopharyngeal aspirates (NPAs) and throat swabs of 325 children, aged from 0.1 to 97.7 months, hospitalized with acute lower respiratory tract infection on the pediatric wards.

### Viral Isolates

Virus cultures were performed via throat swabs with sterile cotton buds and NPA fluids from all subjects within 48 hours of hospitalization. The specimens were preserved in standard transport media and were inoculated on four cell lines (MRC-5 from fibroblast of human fetal lung, Hep-2, A549 from laryngeal carcinoma, and RD cell from rhabdomyosarcoma) for isolation of respiratory viruses. Those culture exhibiting RSV specific cytopathological effects were confirmed by reactions with immunofluorescent antibodies and were stored at −80°C [4].

### Viral RNA Extraction and RT-PCR

The frozen stock viruses were recovered in HEp-2 cells and harvested from cultures when exhibited cytopathological effects in 80% of cells. RSV viral RNA was extracted from 200 µl cultured supernatant using the High Pure Viral Nucleic Acid Kit (Roche Diagnostics, Mannheim, Germany). cDNA synthesis was performed using random primers and Superscript II reverse transcriptase (Invitrogen, Carlsbad, CA). Polymerase chain reaction (PCR) conditions were set according to the manufacturer’s instructions. The PCR products were purified using QIA quick spin columns (Qiagen, Valencia, CA).

### Subgroup Identification and Sequencing

Partial amplification of the RSV G protein gene was performed by PCR, using subgroup-specific primers. Specimens were classified by PCR as RSV-A or RSV-B [18]. The PCR products were 283 bp for human RSV-A and 900 bp for RSV-B. Amplification of the F protein gene was performed using a GeneAmp PCR System 9700 thermocycler (Applied Biosystems Inc.), using the following parameters: 95°C for 10 minutes, followed by 40 cycles of 1 min at 94°C, 1 min at 55°C, and 1 min at 72°C, and finally a 7 min extension at 72°C. PCR primers were similar to the ones in a previous study by Kim *et al* [26]. The N-terminal portion of the F protein gene (nucleotides 3 to 1068 from the N-terminus) was amplified using the RSV-U primer (5′-GGCAAATAACAATGGAGTTG-3′) and the RSV-4R primer (5′-AAGAAAGATACTGATCCTG-3′). The PCR products were sequenced using the BigDye 3.1 Terminator cycle sequencing reagents on an ABI Prism 3730 DNA Analyzer (Applied Biosystems, Forest City, CA).

### Nucleotide and Amino Acid Sequence Analysis

As a measurement of the variability of F protein gene, we calculated the percent identity of RSV F protein gene. Nucleotide and amino acid sequence were multiple aligned by using ClustalW implemented in the MEGA 5 software [27] and the reference strains were A2 (accession number FJ614814) and B1 (accession number AF013254). The jModeltest program was used to estimate the best-fitting nucleotide substitution model [28], [29]. The transition/transversion ratio, gamma-distributed rates, and base frequencies were estimated using the TREE-PUZZLE software version 5.2 [30]. Human HLA class I-restricted CTL epitopes was mapped to the RSV F protein. The position of published CTL epitopes were shaded manually with Genedoc version 2.6.002 (http://www.psc.edu/biomed/qenedoc/). The BIMAS CTL epitope prediction algorithm was used to predict putative CTL epitopes for the F protein [31].

### Phylogenetic Analysis

The subgroup A and B RSV strains were analyzed separately. Nucleotide sequence (position 110–869) of the F protein gene was including in phylogenetic analysis. The nucleotide position is according to FJ614814. Phylogenetic trees were constructed using the neighbor-joining (NJ) and maximum likelihood (ML) methods from the PHYLIP software package (version 3.69, University of Washington, Seattle, WA, USA) [32]. The reliability of the NJ tree topology was statistically evaluated by bootstrap analysis with 1000 replicates. The genetic clusters were identified by using a bootstrap test with a cut-off of 75% [33]. The strains of short sequence length were excluded from phylogenetic analysis. Only one strain was chosen for analysis from each year when more than one strains had identical sequence in the region. There were 70 reference strains including reference strains A2 (accession number FJ614814) and B1 (accession number AF013254) and isolates from previously published studies included for phylogenetic analysis [11], [16], [26], [34]–[36]. The sequences were downloaded from GenBank by BLAST search and the accession numbers of these reference strains were shown in the phylogenetic trees directly.

### Phylodynamic Analysis

The gene evolution rates and population size changes were determined using the Bayesian Markov Chain Monte Carlo (MCMC) method implemented in the BEAST v1.6.2 package (http://beast.bio.ed.ac.uk) [37]. The best substitution model (TrN+G) for the Bayesian analysis was tested using the jModeltest program [28]. The Bayesian skyline plot (BSP) was used to estimate evolution rates and population dynamics under both molecular clock models (strict and relaxed) [38]. The best-fit model was determined using the Bayes factor test in the Tracer program. The MCMC chains were run a sufficient number of times to achieve convergence. In addition, the uncertainty of the parameters was estimated in the 95% highest probability density (HPD). The Maximum Clade Credibility (MCC) tree was constructed using Tree Annotator v 1.6.2.

### Selection Pressure on the F Protein Gene

To determine the selection pressures on the F protein gene in all RSV isolates and subgenera, we estimated the ratio of non-synonymous (*dN*) and synonymous substitutions (*dS*) per site based on the maximum likelihood trees under the TrN substitution model, using the single likelihood ancestor counting (SLAC), fixed effects likelihood (FEL), and internal fixed effects likelihood (IFEL) method with a significance level of 0.05. The FEL method fits codon models to each site independently and performs a likelihood ratio test to evaluate whether a model that assumes equal non-synonymous and synonymous rates (*dN* = *dS*) can be rejected in favor of a model that allows for different *dN* and *dS* rates [39]. Ratios of greater than 1 were considered as evidence for positive selection. Analyses were performed on the Datamonkey website interface (http://www.datamonkey.org) [40].

### Nucleotide Sequence Accession Numbers

The nucleotide sequences of RSV isolates were submitted to GenBank and assigned accession numbers JX477455-JX477594. Prototype reference strains A2 (accession number FJ614814) and B1 (accession number AF013254) were included in nucleotide and amino acid sequence analyses studies.

### Statistical Analysis

Data were analyzed using the JMP software (Version 8). The statistical significance was set at the level of p<0.05.

## Results

### Study Population and Sequence Analysis

Of the 325 RSV isolates, there were 202 RSV-A, 99 RSV-B, and 24 dual infections according to the G protein gene sequences (Table 1). The age of the infected children was 17.9±16.3 (mean ± SD) months. The boy to girl ratio was 1.6∶1. No significant differences in clinical manifestations, such as length of fever and hospital stay, or oxygen use, were noted among the different subgroups. RSV-A predominated during the 2002–2005 and 2006–2008 seasons, whereas RSV-B strains prevailed during the 2005–2006 and 2009–2010 period.

**Table 1:** Clinical presentations of infected patients and yearly distributions.

	RSV-A			RSV-B		Mixed			
Clinical presentation (mean ± SD)									
Age (months)	19.7±17.7			14.8±13.6		16.5±14.8			
Male/female ratio	1.8			1.5		1.2			
Fever duration (days)	3.3±3.0			3.1±3.0		3.1±1.8			
Length of oxygen use (days)	5.3±7.9			5.8±4.5		5.5±4.7			
Length of hospital stay (days)	7.7±7.3			7.5±4.7		8.4±4.2			
C-reactive protein level (mg/dl)	1.9±3.0			1.5±2.6		1.8±3.1			
Yearly distribution	No.	%	No.		%		No.	%	
2000–2001	4	36.4	6		54.5		1	9.1	
2001–2002	4	44.4	4		44.4		1	11.1	
2002–2003	20	76.9	5		19.2		1	3.8	
2003–2004	24	88.9	2		7.4		1	3.7	
2004–2005	23	76.7	7		23.3		0	0.0	
2005–2006	12	42.9	16		57.1		0	0.0	
2006–2007	28	93.3	2		6.7		0	0.0	
2007–2008	20	47.6	14		33.3		8	19.0	
2008–2009	37	62.7	17		28.8		5	8.5	
2009–2010	10	31.3	18		56.2		4	12.5	
2010–2011	20	64.5	8		25.8		3	9.7	
Total	202	60.5	99		32.4		24	7.1	

§ No.-numbers of isolate.

### Nucleotide and Amino Acid Sequence Analysis

**Figure 1:**
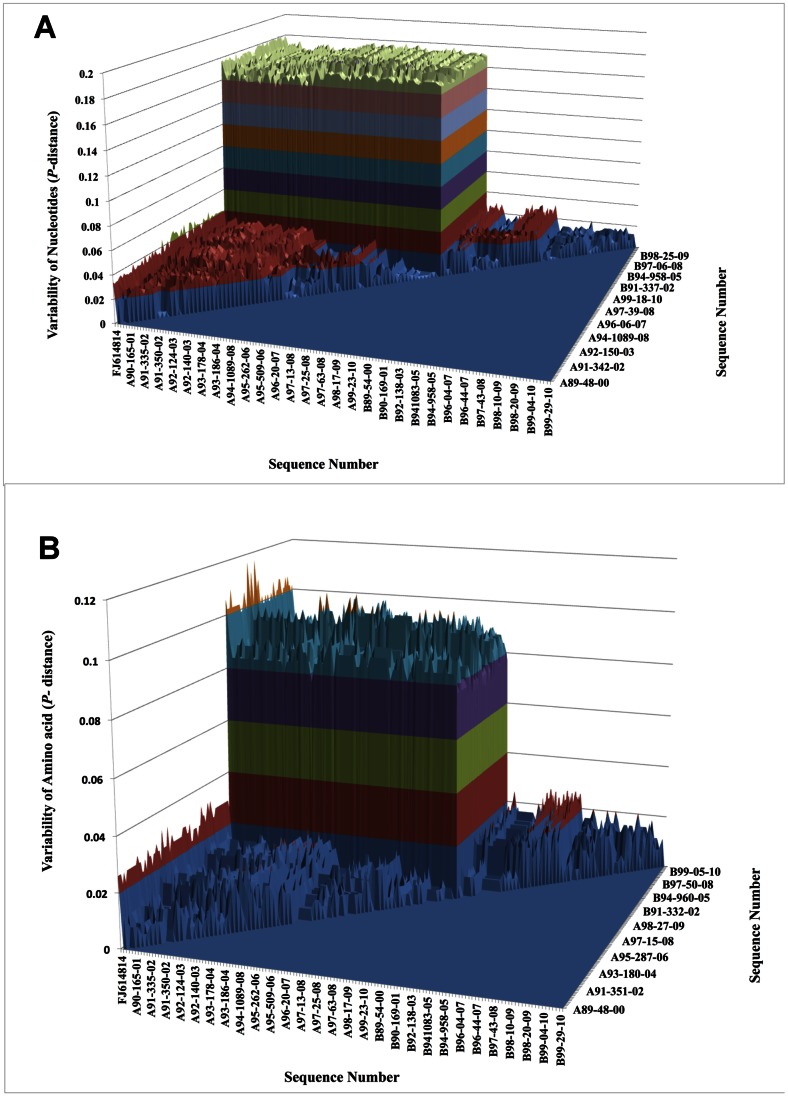
Variability in the fusion protein region of RSV isolated from 2000–2011. 1A shows *P*-distance for nucleotides and 1B shows *P*-distance for amino acid.

The nucleotide *P*-distance is shown in Fig. 1A. The variability was 3.2–4.8% within RSV-A, 0.1–2.3% within RSV-B, and 15.8–18.4% between subgroups. The amino acid *P*-distance is shown in Fig. 1B. The variability was 0–3.3% within RSV-A, 0–2.9% within RSV-B, and 6.9–10.6% between subgroups. Our data suggest that the F protein was more variable for RSV-A than for RSV-B and sequence variation was higher at nucleotide level than at an amino acid level. The overall variability of amino acid of RSV-A and RSV-B compared to reference strains was 3.2–5.0% and 1.7–3.0%. The transition/transversion ratio was 4.88 for RSV-A and 7.12 for RSV-B.

### Phylogenetic Analysis of the F Protein Gene

**Figure 2:**
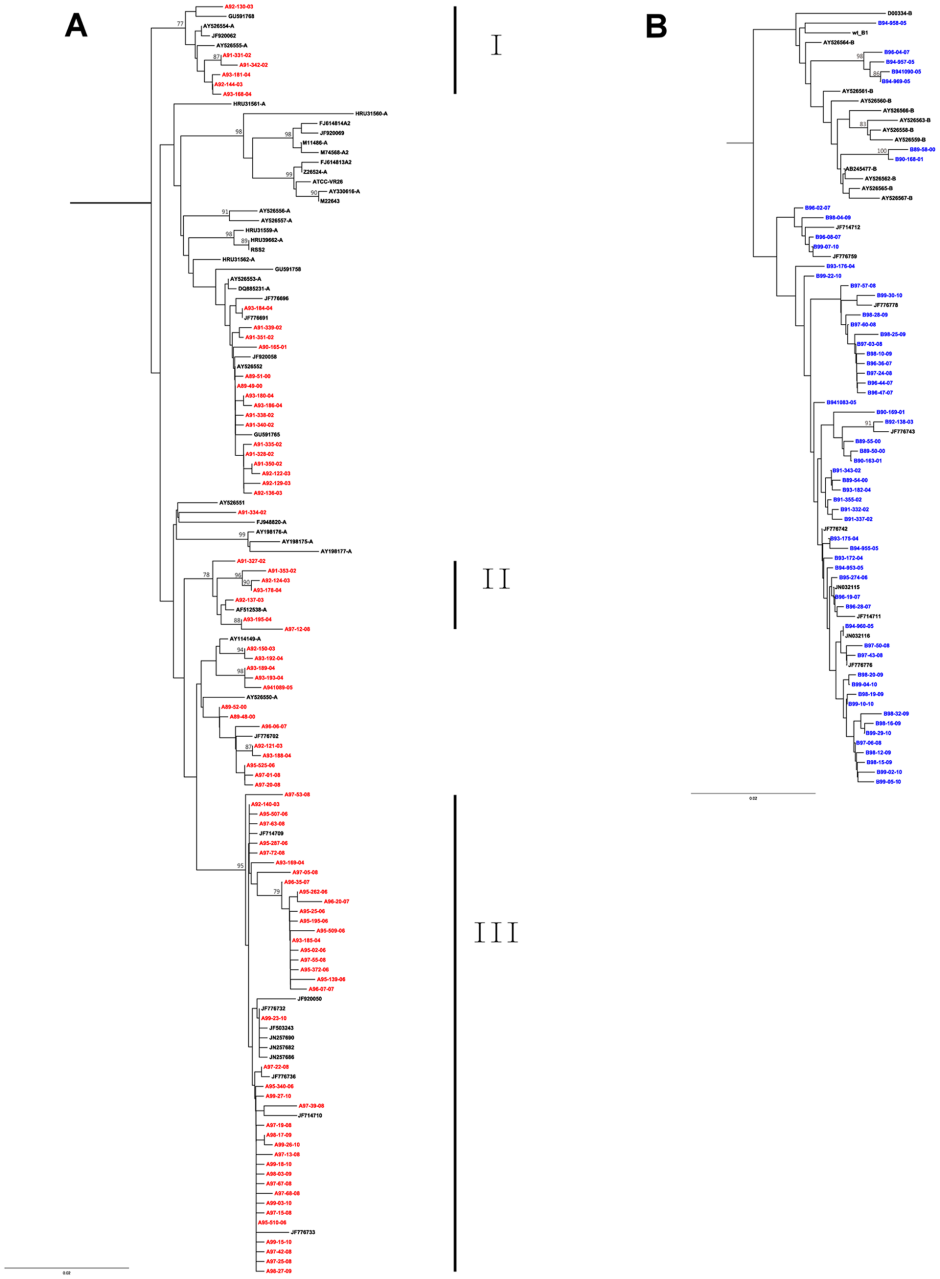
Phylogenetic analysis of the RSV partial F gene (nt 110–869). The tree topology was constructed using the neighbor-joining method. Only bootstrap values greater than 75% (1,000 bootstrap replicates) are shown. Panels A and B show details for RSV-A and RSV-B. Red and blue texts indicate the Taiwanese RSV-A and RSV-B strains, respectively. The scale bar represents the evolutionary distance.

The 141 representative strains for phylogenetic analysis included 83 RSV-A and 58 RSV-B strains (Fig. 2A and 2B). Three clusters were identified among the RSV-A sequences, while no significant grouping was observed among RSV-B sequences. RSV-A cluster I was only noted in the isolates from Years 2002 to 2004. Most strains in cluster II were isolated during 2002 to 2004, with one exception in 2008. RSV-A strains in cluster III were found from 2003 to 2010.

### Amino Acid Variation in Cytotoxic T Lymphocyte (CTL) Epitopes

We list the amino acid variation sites of CTL epitope of RSV- A in the Table 2 and RSV-B in Table 3. Three amino acids that differed between RSV-A and RSV-B (at positions 111, 113, and 114) were found in the HLA-B*57- and HLA-A*01 restricted CTL epitopes. The restricted epitopes were conserved in RSV-A. However, a mutation at position 117 (Y→H) in the HLA-A *01-restricted epitope was more frequent in RSV-B.

**Table 2:** Difference of amino acid in CTL-specific epitopes (HLA-B57, HLA-A*01) between RSV prototype (A2) and Taiwan RSV-A strains.

	HLA-B*57									HLA-A*01									
Amino acid position	106	107	108	109	110	111	112	113	114	109	110	111	112	113	114	115	116	117	118
Prototype A2	R	A	R	R	E	L	P	R	F	R	E	L	P	R	F	M	N	Y	T
RSV-A strains																			
A97-53-2008	**.**	T	**.**	**.**	**.**	**.**	**.**	**.**	**.**	**.**	**.**	**.**	**.**	**.**	**.**	**.**	**.**	**.**	**.**
A95-287-2006	**.**	**.**	**.**	**.**	**.**	**.**	Q	**.**	**.**	**.**	**.**	**.**	Q	**.**	**.**	**.**	**.**	**.**	**.**
A97-72-2008	**.**	**.**	**.**	**.**	**.**	**.**	**.**	**.**	**.**	**.**	**.**	**.**	**.**	**.**	**.**	R	**.**	**.**	**.**
A91-342-2002	**.**	**.**	**.**	**.**	**.**	**.**	**.**	**.**	**.**	**.**	**.**	**.**	**.**	**.**	**.**	**.**	**.**	H	**.**
A97-20-2008	**.**	**.**	**.**	**.**	**.**	**.**	**.**	**.**	**.**	**.**	**.**	**.**	**.**	**.**	**.**	**.**	**.**	**.**	K
A89-48-2000	**.**	**.**	**.**	**.**	**.**	**.**	**.**	**.**	**.**	**.**	**.**	**.**	**.**	**.**	**.**	**.**	**.**	**.**	K

§ A: Alanine, E: Glutamic acid, F: Phenylalanine, H: Histidine, K: Lysine, L: Leucine, M: Methionine, N: Asparagine, P: Proline, Q: Glutamine, R: Arginine, T: Threonine, Y:Tyrosine.

* Prototype A2: accession number FJ614814.

**Table 3:** Difference of amino acid in CTL-specific epitopes (HLA-B57, HLA-A*01) between RSV prototype (B1) and Taiwan RSV-B strains.

	HLA-B*57									HLA-A*01									
Amino acid position	106	107	108	109	110	111	112	113	114	109	110	111	112	113	114	115	116	117	118
Prototype B1	R	A	R	R	E	A	P	Q	Y	R	E	A	P	Q	Y	M	N	Y	T
RSV-B strains																			
B-96-28-2007	**.**	**.**	**.**	**.**	**.**	**.**	S	**.**	**.**	**.**	**.**	**.**	S	**.**	**.**	**.**	**.**	**.**	**.**
B-99-20-2010	**.**	**.**	**.**	**.**	**.**	**.**	**.**	K	**.**	**.**	**.**	**.**	**.**	K	**.**	**.**	**.**	**.**	**.**
B-99-05-2010	**.**	**.**	**.**	**.**	**.**	**.**	**.**	**.**	H	**.**	**.**	**.**	**.**	**.**	H	**.**	**.**	H	**.**
B-96-02-2007	**.**	**.**	**.**	**.**	**.**	**.**	**.**	**.**	**.**	**.**	**.**	**.**	**.**	**.**	**.**	**.**	S	**.**	**.**
B-96-04-2007	**.**	**.**	**.**	**.**	**.**	**.**	**.**	**.**	**.**	**.**	**.**	**.**	**.**	**.**	**.**	**.**	**.**	H	**.**
B-94-969-2005	**.**	**.**	**.**	**.**	**.**	**.**	**.**	**.**	**.**	**.**	**.**	**.**	**.**	**.**	**.**	**.**	**.**	H	**.**
B-94-957-2005	**.**	**.**	**.**	**.**	**.**	**.**	**.**	**.**	**.**	**.**	**.**	**.**	**.**	**.**	**.**	**.**	**.**	H	**.**
B-94-1090-2005	**.**	**.**	**.**	**.**	**.**	**.**	**.**	**.**	**.**	**.**	**.**	**.**	**.**	**.**	**.**	**.**	**.**	H	**.**
B-93-176-2004	**.**	**.**	**.**	**.**	**.**	**.**	**.**	**.**	**.**	**.**	**.**	**.**	**.**	**.**	**.**	**.**	**.**	**.**	I

§ A: Alanine, E: Glutamic acid, F: Phenylalanine, H: Histidine, I: Isoleucine, K: Lysine, L: Leucine, M: Methionine, N: Asparagine, P: Proline, Q: Glutamine, R: Arginine,

S: Serine, T: Threonine, Y:Tyrosine.

* Prototype B1: accession number AF013254.

### Selection Pressure on the F Protein

The selection pressure on the RSV isolates examined in this study was estimated using the *dN*/*dS* ratio (Table 4). The mean *dN*/*dS* ratio was 0.102 in all RSV isolates, 0.093 in RSV-A and 0.137 in RSV-B. No positive selection sites were found for RSV-A, whereas 1 positive selection site was observed for RSV-B (position 125; L→P).

**Table 4:** Selection sites detected in RSV fusion protein genes.

	Positively selected sites			No. of negatively selected sites			*d_N_*/*d_S_*
	SLAC	IFEL	FEL	SLAC	IFEL	FEL	
RSV isolate	None	None	103 (0.03)*	33	37	70	0.102
RSV-A	None	None	None	9	7	23	0.093
RSV-B	None	125 (0.02)*	None	1	3	9	0.137

§ p-value <0.05 are indicated by*. Selection site detection was performed on the Datamonkey website (http://www.datamonkey.org) with the single likelihood ancestor counting (SLAC), fixed effects likelihood (FEL), and internal fixed effects likelihood (IFEL) methods.

### Evolutionary Rate and Phylodynamics of RSV

**Figure 3:**
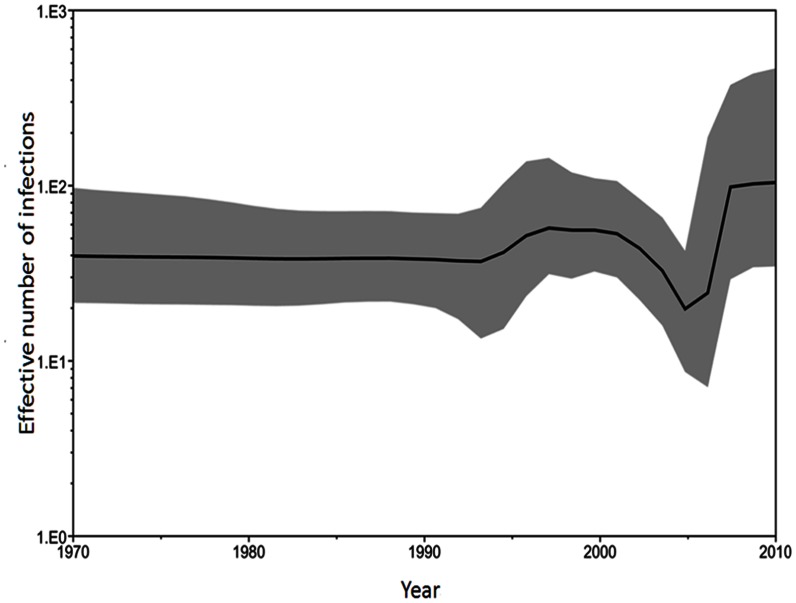
Bayesian skyline plot derived from alignment of the RSV partial F gene sequences in Taiwan. The x axis is in units of years before 2010, and the y axis represents the effective population size of the virus (i.e. the number of genomes effectively contributing to the next generation). The thick solid black line is the estimated median, and the pale gray areas show the upper and lower bounds of the 95% HDP interval.

The evolutionary rate of the RSV F protein was 1.00×10^−3^ substitutions/site/year, and the 95% HPD was 0.823–1.183×10^−3^. The nucleotide substitution rates for the 3rd codon position (synonymous: 2.363, 95% HPD 2.233–2.489) were significantly higher than for codon positions 1 and 2 (nonsynonymous: 0.338, 95% HPD 0.250–0.437; 0.30, 95% HPD 0.208–0.399, respectively). Time to most recent common ancestor (TMRCA) for RSV-A and RSV-B in this study was estimated for the years 1968 (95% HPD, 1960–1976) and 1990 (95% HPD, 1994–1986). Bayesian skyline plot (BSP) models were used to estimate changes in epidemic history and evolutionary dynamics of RSV infection over time. Uncertainty in the estimated parameters was evaluated using 95% HPD intervals. The BSP describing the effective population size of RSV is shown in Figure 3. The genetic diversity of the viral population remained steady until 1995. Subsequently, the slight increase the population size during 1995 to 2000, followed by slowly declined until 2005, when it began a significant increase that lasted till 2010.

## Discussion

To date, molecular evolution and phylogenetic analyses of RSV spanning successive seasons have not been reported for viruses circulating in Taiwan. In this study, we found that both RSV subgroups co-circulate in Taiwan and the genetic diversity of the F protein was more pronounced in subgroup A. A mutation at position 117 was more frequent in the HLA-A *01-restricted epitope in subgroup B. Positive selection was observed at site 125 in subgroup B. Phylodynamic patterns showed increased evolution of RSV in recent 5 years. Previous studies have suggested that illness severity is not linked to subgroups or genotypes but to the abundance of RSV in the nasopharyngeal aspirate [8], [41]. We also found no difference in the severity of disease caused by RSV-A and RSV-B.

Several community-based studies have revealed that viruses from both antigenic subgroups may co-circulate with one predominate during individual epidemic periods; and the dominant viruses can change genetically from one year to the next and replacement of genotypes in successive seasons [17], [18]. In accordance with these findings, we observe that RSV-A and RSV-B co-circulated and were dominant separately in Taiwan during certain years. Besides, our analysis of the genetic variability of the RSV F protein reveals evidence of shifts in the predominant three genetic clusters during 11 years and indicates genetic displacement of RSV-A. This process is characterized by the disappearance of several genotypes and the emergence of new genotypes within the same community, as observed over nine consecutive epidemics in Korea [26].

Antigenic variation in RSV may affect infectivity, as the RSV F and G proteins are targets for neutralizing antibodies [12], [42]. The RSV F-protein is highly conserved between subgroups with more than 90% amino acid sequence identity and it is not easy to undergo positive selection over the complete protein [11], [43]. To better understand the epidemiology and mechanisms of RSV reinfection, we therefore chose to study the F protein genes over an 11-years period in a localized geographical region. Despite the conserved nature of the F protein, the variation does occur between different strains and between the subtypes in our study as well as previous study [34]. We found that the transition/transversion ratio was 4.88 and 7.12 for subgroups A and B, which are similar to the values obtained in a previous report [16]. Furthermore, previous studies have suggested that mutations in the F protein gene are responsible for immunity to the anti-RSV drug Palivizumab in a small number of patients [44].

Virus-specific CTL play a major role in the clearance of RSV infection. We discovered 3 amino acid changes (at positions 111, 113, and 114) in the HLA B*57- and HLA-A*01-restricted epitopes of RSV-B but not in RSV-A. Our data are similar to those reported from a study in South Africa [16]. No positive selection site was found on CTL epitopes in Taiwan strains, suggesting that immune selection may not occur.

In our study, phylogenetic analysis showed that the genetic diversity of the F protein was more pronounced in RSV- A than in RSV-B, this is similar to other studies [16], [26]. Three RSV-A clusters in our study, containing most of the Taiwan strains, were identified. Each cluster comprised strains isolated during a different time period. These results imply that before 2004, RSV-A infections were involved in several small epidemics and only very limited numbers of strains evolved and re-emerged in subsequent years, as seen in cluster II. After 2005, the circulating RSV-A strains were quite different from those of the previous years; these strains continued evolving through 2010. In contrast to the phylogeny of RSV-A sequences, no significant clustering was found among the RSV-B phylogenies. This result is concordant with the estimation by TMRCA that RSV-B might have a shorter evolutionary history in Taiwan than RSV-A. The Taiwanese RSV-B strains spread over the phylogenetic tree, regardless of the isolation time. It suggests that the RSV-B strains were continually circulating in Taiwan.

In a previous study in New Zealand, the mutation rate calculated for the RSV-B G-gene was significantly higher than that of RSV-A, indicating that RSV subgroups exhibit different patterns of evolution, with subgroup B viruses evolving faster than those of subgroup A [45]. We found the evolutionary rate of the RSV F protein was 1.00×10^−3^ substitutions/site/year in our study, which was slower than that of the G protein gene (1.83×10^−3^) by Zlateva *et al* [46] and faster than whole genome (6.47×10^−4^) by Tan *et al* [47].

BSP is a very powerful tool to estimate changes in epidemic history and evolutionary dynamics of viral infection over time. The evolution rate of RSV in Taiwan had very little change before 2000, then slowed down between 2000 and 2005, and significantly evolved faster after 2005. The BSP pattern was in agreement with the phylogenetic analysis. Furthermore, comparison of the BSP and phylogenetic data suggests that the increase in the infected population was due to the co-circulation of RSV-A F protein cluster III and RSV-B type viruses.

In conclusion, RSV-A infections were involved in several small epidemics before 2004 and only very limited numbers of strains evolved and re-emerged in subsequent years in Taiwan. After 2005, the circulating RSV-A strains were different from those of the previous years and continued evolving through 2010. Phylodynamic pattern showed the evolutionary divergence in RSV increased significantly in recent 5 years. The evolutionary divergence in RSV is probably related to differences in immune protection in the hosts. Phylodynamic patterns showed an increase in the evolution of RSV in recent years, suggesting that monitoring of the gene will be necessary. Our results may provide important information for vaccine design and epidemiological studies.
